# A Game of Hide and Seek: Expectations of Clumpy Resources Influence Hiding and Searching Patterns

**DOI:** 10.1371/journal.pone.0130976

**Published:** 2015-07-08

**Authors:** Andreas Wilke, Steven Minich, Megane Panis, Tom A. Langen, Joseph D. Skufca, Peter M. Todd

**Affiliations:** 1 Department of Psychology, Clarkson University, Potsdam, NY, United States of America; 2 Department of Mathematics and Computer Science, Clarkson University, Potsdam, NY, United States of America; 3 Department of Psychology, University of Lyon, Lyon, France; 4 Department of Biology, Clarkson University, Potsdam, NY, United States of America; 5 Cognitive Science Program, Indiana University, Bloomington, Indiana, United States of America; University of Nottingham, UNITED KINGDOM

## Abstract

Resources are often distributed in clumps or patches in space, unless an agent is trying to protect them from discovery and theft using a dispersed distribution. We uncover human expectations of such spatial resource patterns in collaborative and competitive settings via a sequential multi-person game in which participants hid resources for the next participant to seek. When collaborating, resources were mostly hidden in clumpy distributions, but when competing, resources were hidden in more dispersed (random or hyperdispersed) patterns to increase the searching difficulty for the other player. More dispersed resource distributions came at the cost of higher overall hiding (as well as searching) times, decreased payoffs, and an increased difficulty when the hider had to recall earlier hiding locations at the end of the experiment. Participants’ search strategies were also affected by their underlying expectations, using a win-stay lose-shift strategy appropriate for clumpy resources when searching for collaboratively-hidden items, but moving equally far after finding or not finding an item in competitive settings, as appropriate for dispersed resources. Thus participants showed expectations for clumpy versus dispersed spatial resources that matched the distributions commonly found in collaborative versus competitive foraging settings.

## Introduction

Children encountering the venerable game of *Battleship* may try out the simple strategy of positioning all their ships—the carrier, sub, patrol boat, and so on—in one big clump, perhaps cleverly offset from the center of the board to provide their seeking opponent an extra tough challenge. With time (and possibly some crushing naval defeats), they may adopt a different tactic: Spreading out their valuable resources across the space, making it harder for seekers to hit a jackpot. In doing so, they are mirroring the food-hiding strategies of many animal species. They are also, as we argue here, revealing assumptions that humans have for how resources are typically distributed in space.

People have been shown to expect to encounter particular types of events in sequential clusters over time—for instance, a basketball player’s successful shots coming in streak, or a succession of berries found on a bush until they run out [1–3]. In this paper we move from the one-dimensional temporal case to investigate when people will expect clumpy or patchy resources in two-dimensional space. We do this using a simplified version of the *Battleship* game in which players both hide and search for resources in a spatial grid, varying whether they are working collaboratively (“hiding” resources so that others can find them) or competitively (hiding resources to be difficult to find). When searching, we predict individuals will follow their expectations for how the environment will be patterned, while when hiding, they will respond to the expectations they believe others have. In other words, to discover how people search for spatially distributed resources, and thus how they expect resources to be distributed in the environment in different situations, we use an experimental task that involves both searching, which people have some experience with, and hiding resources, which individuals may not often do outside of game contexts but which nonetheless gives us a window into their thinking about resource patterns. In the following sections we place these questions in the context of animal hoarding and foraging strategies and past research on human hiding and seeking, before describing the game and results and discussing the implications regarding human expectations of environment structure.

### Seeking: Search on resource distributions in natural environments

Many animal species including humans search for resources in their environment in ways that are well-adapted to resources coming in clumps or patches [4,5]. This can be seen in the use of patch-leaving rules for continuing to exploit resource patches as long as they are sufficiently profitable [6–9], as well as the ubiquitous area-restricted search tactics that make organisms focus search near where they have already found resources [10,11]. Not surprisingly, the explanation for these types of search behavior can be found in the statistical resource structure and ecology of natural environments: Resources (such as food and mates) are mostly distributed in patches in natural environments, whereas randomness or dispersion are less common [12–14]. Patchy resource distributions can arise due to habitat preferences of plants and prey animals, predator avoidance, or reproductive opportunities leading organisms to gather in a particular region (e.g., [15]). Spatial dispersion does occur in some natural situations, however, including for tropical forest trees, nesting birds, and territorial animals, driven by infectious pathogens, predator search behavior, or interspecific competition, as we discuss in the next section [16–18]. Theoretical and empirical findings in behavioral ecology have shown how the underlying mechanisms involved in foraging behavior were shaped by the distribution of resources in the environment (e.g., [6,7,19–21]).

**Fig 1:**
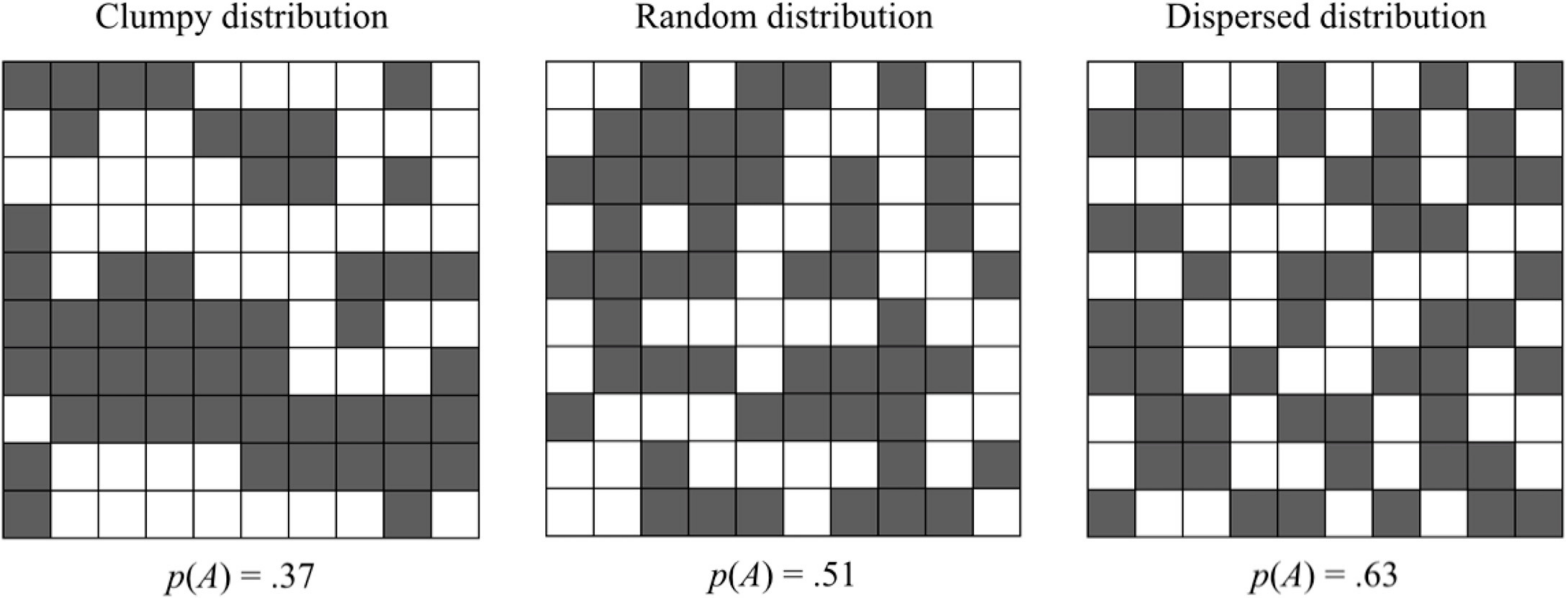
Spatial resource distributions. Three 10×10 resource grids of 50 white squares and 50 black squares differing in their underlying spatial distribution determined by alternation rate *p*(*A*). Adapted from Falk & Konold (1997).

Evidence that the human mind evolved to often expect resources in clumps or patches can be seen in people’s common perceptions that random sequences of events are clustered into “runs” or streaks of being “hot” (e.g., [2,3,22]; cf. [23]). Furthermore, people often perceive spatial clumps in random 2-dimensional resource patterns. Falk ([24], described in [25]) showed this with a set of 10×10 resource grids in which half of the 100 squares were empty (colored white) and half had a resource (colored black). Each pattern was generated according to an alternation rate *p*(*A*) that specified the probability that the next square would differ from the previous one (going left-right and top-bottom). Whereas grids with an alternation rate *p*(*A*) near 0.5 are least predictable (and hence most random in at least a colloquial sense), lower alternation rates [*p*(*A*) < .5] create clusters of empty or full squares and higher alternation rates [*p*(*A*) > .5] lead to more dispersion (see Fig 1). But when asked to rate the randomness of the visual grid arrangements, participants did not give the highest ratings to grids with alternation rate near 0.5—they chose grids which were more dispersed [with *p*(*A*) around .60–.65]. As in one-dimensional sequences, the least predictable random grid arrangements [*p*(*A*) = .5] were perceived as having clusters of resources (see [25]; see also [26] for search behavior in smaller grid sizes).

### Hiding: Food hoarding in animals

Food hoarding, defined as hiding food in locations that are hidden from conspecifics but that the individual can return to later, is a strategy that enables animals to increase the likelihood of having food in times of scarcity [27]. In *larder hoarding* species, a single cache location is typically used, reducing the memory load on the hoarder for remembering where to find the cache in the future but increasing the potential loss if pilferers find the cache. To avoid the latter problem, *scatter hoarders* put smaller amounts of resources in multiple locations, but thereby increase their potential memory costs. Because we are interested in human expectations regarding spatial resource distributions as revealed in how people hide and find multiple resources in multiple locations, we focus on scatter hoarding here.

A major adaptive challenge that arises with food caching is the presence of other animals that may steal a hoarder’s hidden food. To reduce cache loss from competitors, animals have to use effective resource hiding strategies. Ideally, such an effective strategy should both increase the hoarder’s odds of finding the caches later, but at the same time decrease the odds of other animals finding the stashed food supplies (see [28]). When food is limited and there is the possibility of pilferers (including thieving conspecifics) finding one’s food caches, scatter hoarders try to improve their chances of recovering stored food by caching larger amounts of food (e.g., [29,30]), moving the food away from its source to hide it in dispersed caches (e.g., [31,32]), or avoiding caching when in the presence of conspecifics (e.g., [33,34]). These responses, however, often impose additional costs on the scatter hoarders; for instance, using more distant food caches can reduce the amount of time that is available for food collection, and additional cache locations can increase the burden on memory when aiming to retrieve these locations in the future. Previous studies suggest that the strategies scatter hoarders use depend on factors such as the presence of conspecifics (e.g., [35]), the age or social status of the animal (e.g., [36]), and the amount of food available (e.g., [37]).

The strategy of manipulating the density and dispersal of caches so as to minimize the foraging efficiency of pilferers is used by many species. Studies with fox squirrels, for instance, found that the mean survival time of buried walnuts from pilfering (i.e., how long they remained available to be found by the original hoarder) tended to increase with decreasing nut density [28]. Similarly, marsh tits store seeds at a density that minimizes cache loss [38] and agoutis cache seeds further away from their source and in lower densities when there is increased pressure from food competitors [39]. But beyond these findings that scatter-hoarding animals typically store food at a particular density, the question arises whether there is a specific *type* of distribution that might minimize cache loss.

As Male and Smulders [30] lay out, a scatter-hoarding animal could hide its food in a *clustered* distribution (where the presence of one resource cache increases the probability of finding another nearby), a *random* distribution (where resources occur independently from one another), or a *hyperdispersed* distribution (that is, more dispersed than random, where the presence of one resource decreases the probability of finding another nearby, as in a uniform distribution). As indicated in the previous section, animal species often forage more efficiently on clumped resources than on more dispersed ones, fitting with the clumpy or patchy distribution of most resources in nature [2,15]. Male and Smulders [30] for instance investigated how quickly seed-eating birds found food items from artificial resource distributions that were either uniform (hyperdispersed), random, or clustered. After three days, an average of 56% of the artificial caches remained in the hyperdispersed distribution, while only 40% were still present in the clustered distribution. Hyperdispersing resources was also found to lead to a reduction in cache loss in studies including birds in the laboratory [40], fish [41], cattle [42], and ants ([43]; see [30] for a brief review). This may be because pilferers are using a win-stay strategy appropriate for (common) clustered resources, rather than a win-shift strategy more fitting for hyperdispersed resources. Consequently, because more dispersed resource distributions increase competitors’ search time and take longer to harvest, scatter hoarders may benefit by hiding their food caches from pilferers in such spatially dispersed patterns.

Dispersing food items, however, comes at a cost for the hoarding animal in terms of their main goal: finding their cached food for their own later consumption. For many scatter hoarder species, the time between storage in plentiful periods and retrieval in times of scarcity can be multiple months [44]. To the extent that the hoarder relies on memory to guide its retrieval search, a more dispersed pattern requires the hoarder to remember more spots of smaller size than a clustered pattern where resource items are often cached near each other, necessitating only a few remembered locations of clusters plus area-restricted search to find much of the stored food within each cluster. Using (hyper)dispersion of resources can thus be costly in terms of the cognitive capacity required to keep the detailed location information in memory. In line with this, many studies find that scatter-hoarding species show greater spatial learning and memory performance than similar non-hoarding species (e.g., [45]), supporting a correlation between food caching and spatial memory, including hippocampal volume, particularly when short-term retention is involved (e.g., [46,47]; for a review see [48]).

### Hiding and seeking behavior in humans

Research on hiding and searching strategies in humans that focuses on the role of spatial resource distributions is scarce, but the few studies done so far provide results that are consistent with the animal literature. These studies mostly adopt a scatter-hoarding perspective, involving hiding and searching for multiple items rather than just one (but see [49] for a minimal hide/seek game with one resource). Talbot et al. [50], instructed adults to either hide or search for three items among nine hiding locations (items were hidden by others; participants were not asked to re-find the items they hid, in contrast to the studies in this paper). Participants moved further away from their starting location and dispersed their choices more when hiding objects than when searching for objects hidden by others, though experience in the hiding task increased the distance and the amount of dispersion that participants showed when searching for the hidden locations. Participants’ searching and hiding behavior was unaffected by the kind of environment they searched in (e.g., a virtual vs. real-world environment), but their reported verbal strategies indicated that participants hid objects strategically in order to make it difficult for other searchers to retrieve these items. Recent studies with more complex search environments (e.g., rooms filled with furniture, dark corners, and windows) replicated these findings [51]. These studies suggest that overall, adults believed that others searching would have expectations of clumpy resources and so hid the resources accordingly in a dispersed manner, but then the same individuals were influenced themselves by those expectations of clumps when they were in the searching role and did not turn them off sufficiently to effectively find dispersed items.

Developmental psychologists have looked at similar hiding and searching behaviors in children. Cornell and Heth [52] observed the search patterns of 6–8 year old children when searching for 20 marbles among 100 possible hiding locations in a room. (Children both hid and then searched for the marbles in 100 envelopes placed in clusters of three around the perimeter of a large cluttered play room.) Young children tended to concentrate their hiding locations in one sector of the room, typically located away from the entrance. With increasing age, however, they started to disperse their hiding locations across multiple areas in the room, and using less clustering and more scattering. The researchers suggested that this developmental change in hiding patterns could be due to the conflicting goals of selecting sites that minimize pilferage by others and that are easily remembered. Older children may start to disperse their hiding sites as they can remember multiple locations better than younger children. When the children had to find the marbles that another person had previously hidden, younger children showed more evidence of a win-stay strategy to search through resource clusters (cf. [53]), that is, looking for another marble nearby (“staying”) after they found a marble in a particular location (“winning”). This is half of the win-stay lose-shift search strategy (search again nearby after finding a resource, and move further away after not finding a resource) which like the related area-restricted search strategy is appropriate for clumpy distributions, and which has also been shown by chimpanzees in comparable search tasks [54]. Older children more often used a win-shift strategy, but not enough to increase their finding probability above chance levels. That is, as for adults, children were often influenced in their searching by an expectation of clumpy resources, and did not sufficiently avoid such influences even when they themselves had experience hiding resources in a more dispersed fashion.

In sum, this past work suggests that human children and adults have strong default expectations of resources being in clumps or patches, and they search for hidden items as though influenced by this expectation even when they have experience hiding items in more dispersed, less patchy patterns.

### Hypotheses

As indicated in the overview above, resources that are meant to be hidden from discovery and use or consumption by others (in what we call competitive foraging settings) are often located in hyperdispersed patterns, because of increased vulnerability when found in spatial clusters (though there are exceptions where the benefits of clustering outweigh the costs, e.g. for groups of animals hiding from predators). On the other hand, resources that are intended to be found and used (in what we call collaborative foraging settings) are often agglomerated in clusters, as in fruit in patches of bushes or trees, males of many species gathering in groups called leks to display to female potential mates, retail shops located near each other to attract more customers, or cultural resources being put into collections in libraries or museums to facilitate browsing and search (see [55] for a comparison). Here we ask whether humans’ expectations regarding the spatial distribution of resources in competitive versus collaborative settings match these common environmental structures. Given that individual participants may not have much experience in hiding resources (whether or not those resources are to be found by others), we are not proposing that hiding resources is a particularly common or ecologically valid task for modern humans (or our ancestors); rather we are using hiding and seeking behavior elicited in the laboratory to uncover those distribution expectations that we are interested in. This leads to the following hypotheses about the expectations that humans will have for spatial resource distributions in competitive and collaborative settings and the hiding and seeking behaviors corresponding to those expectations:

#### Hypothesis 1

In a competitive foraging situation, participants will hide resources in a hyperdispersed rather than random pattern [*p*(*A*) > .5] so that they are more difficult for others to find.

#### Hypothesis 2

In a collaborative foraging situation, participants will hide resources in a clustered pattern [*p*(*A*) near 0] so that they are easier for others to find.

#### Hypothesis 3

When participants must themselves later retrieve the resources they hide from others, they will hide resources in a more clustered manner [lower *p*(*A*)].

#### Hypothesis 4

When seeking resources hidden by others, participants will use a win-stay lose-shift strategy appropriate for clumpy resources more when they are in a collaborative foraging situation than in a competitive foraging situation.

That is, we predict that because people expect resources to come in patches in typical situations, they will look for them using strategies appropriate for patches especially in collaborative settings (Hypothesis 4); when they are hiding resources from others, they assume those others can look for them using the same clumpy-resource-appropriate strategies, so they hide them in ways that do not look clumpy (even if they are more predictable as a result) (Hypothesis 1), though possibly less so if they must also remember where to retrieve those resources from later (Hypothesis 3); while in collaborative settings, hiders will count on the clumpy search patterns of others and so position the resources in easy-to-find patches (Hypothesis 2).

## Methods

**Fig 2:**
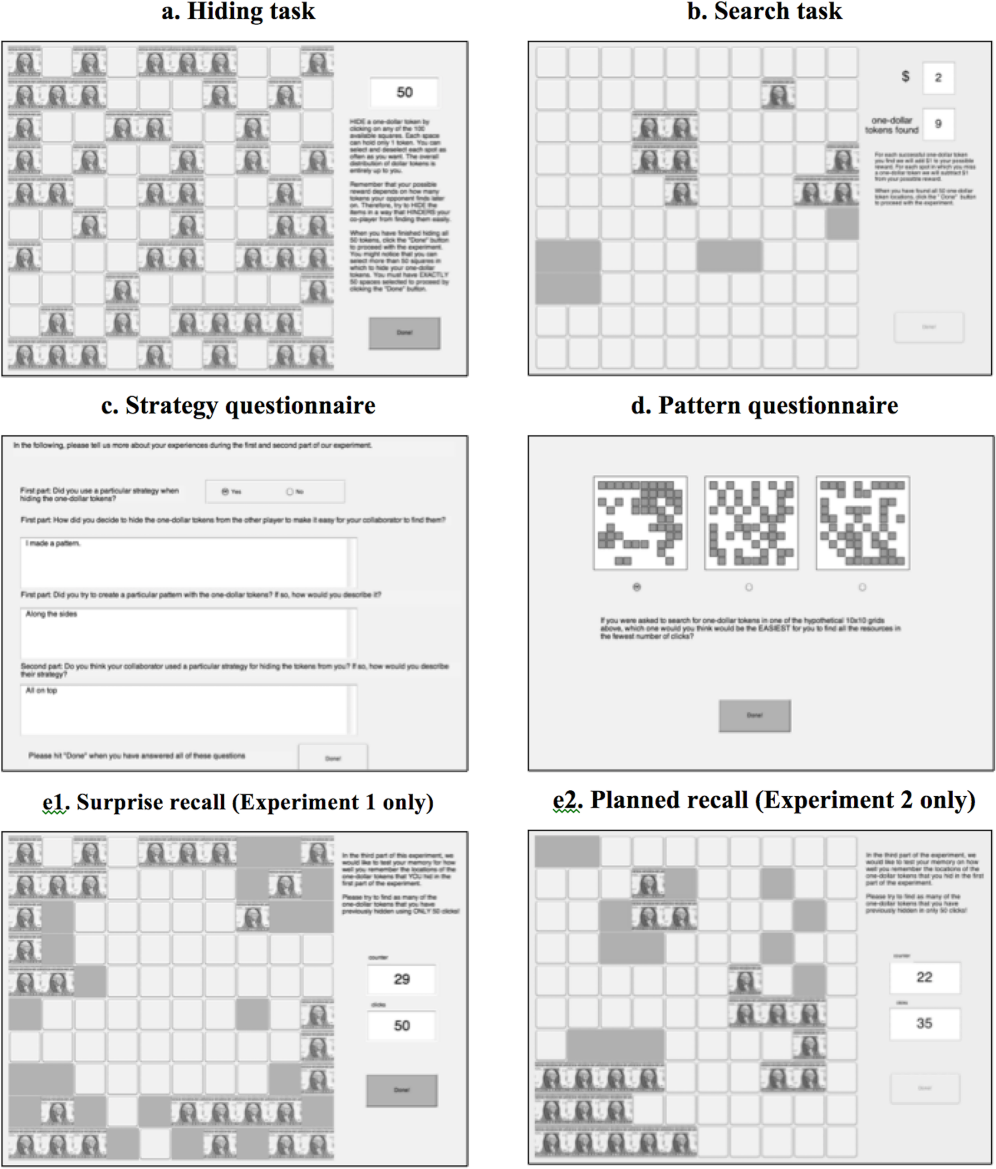
Overview and order of experimental tasks in the computerized hide and seek game.

To study the mechanisms and patterns used by humans in collaborative and competitive caching/foraging situations, we had participants hide and search for resources in a 2-dimensional grid space (coded in MatLab R2011b). Fig 2 gives an overview of the five distinct parts of the experiment, which each participant completed in the same order.

In the first stage of the experiment, participants were asked to hide 50 resources (dollar bill images) in a 10×10 square grid containing 100 potential resource locations. They were instructed that the next participant being tested in the laboratory would search through their hiding pattern and that their later payoff for this part of the experiment depended on the success of that very same person searching for their hidden resources (positively or negatively depending on condition, as explained below). Clicking a grid position with the mouse cursor filled that location with a dollar image if it was empty; if it already had a dollar image in it, that location would instead return to being empty. Participants were free to select (and deselect) spots for as long as they wanted, but could only move on to the next part of the experiment by clicking on the “Done” button when exactly 50 resources were hidden. A counter in the upper right corner indicated the number of resources currently hidden.

In the second stage of the experiment, participants were asked to search for 50 resources on a new 10×10 resource grid. They were told that the previous participant in this experiment had hidden the resources with instructions that their payoff for this part of the experiment depended (again, positively or negatively) on how successful the current participant would be at finding those hidden resources. Participants clicked the grid locations one at a time with immediate feedback after each selection as to whether that location contained a resource (i.e., a dollar bill) or not (i.e., an empty green square). The search task ended once participants found all 50 tokens that were hidden in the grid. (We always had hiding come before searching so that participants’ hiding behavior would not be influenced by the hidden resource patterns created by others.)

After the search task was over, participants completed the third and fourth part of the experiment. The third part contained a strategy questionnaire in which participants answered 1) how they decided to hide the resources from the other player, 2) if they tried to create a particular pattern with the resources, and 3) if they thought their opponent in the second stage used a particular strategy to hide the resources from them. The fourth part contained a visual pattern questionnaire in which participants saw three successive sets of three 10×10 grids and answered one question about each set. Within each set (in random order), one grid had an alternation probability *p*(*A*) = .30 (i.e., a clumpy distribution), one had *p*(*A*) = .50 (a random distribution), and one had *p*(*A*) = .70 (a hyperdispersed distribution). Participants chose which of the three grids shown they thought to be 1) *hardest* for their opponent to find resources in, 2) *easiest* for their opponent to find resources in, and 3) most *random* in its distribution.

We ran two versions of this experiment to investigate the tradeoffs that participants make between creating hiding patterns that are easy for them to remember but also easy for pilferers to exploit, and those that are difficult to exploit but also difficult to remember. The two versions differed only in the fifth stage of the experiment, where participants were asked to recall all the locations of the 50 resources that they hid in the first stage of the experiment. In Experiment 1 this recall task was not mentioned earlier to participants (thus, it was a *surprise* recall, and participants could be assumed to not take their own memory requirements into account during the hiding stage). In Experiment 2 participants were instructed in the first stage that there would be a recall stage at the end of the experiment (a *planned* recall, so participants could be assumed to take their memory requirements into account during hiding in this case). Participants made 50 clicks on an initially blank grid and received immediate feedback after each click showing their success or failure at finding their own earlier hiding locations.

In each experiment, recruited participants were counterbalanced to participate in one of two experimental conditions: a *competitive* or a *collaborative* condition. In the competitive condition, participants were told to hide their resources in such a way that it would be most difficult for the other player to find them (and that their payoff would depend on how *little* success the other player has). In the collaborative condition, participants were asked to hide resources so that their opponent would have no difficulty finding them (and that their payoff would depend on how *much* success the other player has). Likewise, in the search task, participants were paired with the previous person from the same condition and were told that that person hid the resources to make them difficult to find (competitive condition) or easy (collaborative condition). This is similar to the competitive and cooperative conditions of [26], but differs in our much greater number of resources and sequential search with feedback, which are features of many real foraging settings. (While this strategic interaction bears some resemblance to public goods games in that players are making resources available, or not, to others, here players do not decide *whether* to make some amount of resources available, but rather, after being told to make the resources available (findable) or not, they decide *how* to make those resources available or unavailable to the next player. In that sense it is more similar to coordination games [49].)

After all participants had been run in each experiment, we randomly drew 4 participants via a lottery and paid them their combined earnings from the hiding and searching tasks. For the search task, in both conditions participants received $1 for each resource they found, but also lost $1 for each empty location they clicked on. For the hiding task, in the competitive condition participants were paid for successfully hiding resources from their opponent, with their payoff set at $50 minus the opponent’s searching payoff. In the collaborative condition, participants were paid for positioning resources so their opponent was able to find them, with their payoff set equal to the opponent’s searching payoff. The mean payoff per selected participant was $45.50 in the competitive condition and $89 in the collaborative condition.

### Participants

In both experiments, Clarkson University undergraduates participated for course credits (Experiment 1: *N* = 158, 79 women, 79 men, mean age 19 years, range 16–22 years; Experiment 2: *N* = 135, 59 women, 76 men, mean age 19 years, range 16–45 years). Participation in both experiments was approved by Clarkson University’s Institutional Review Board (#12–24). Participants provided written consent. Participating minors had to present a signed parental permission form.

## Results

### What spatial distributions do participants use to hide their resources?

**Fig 3:**
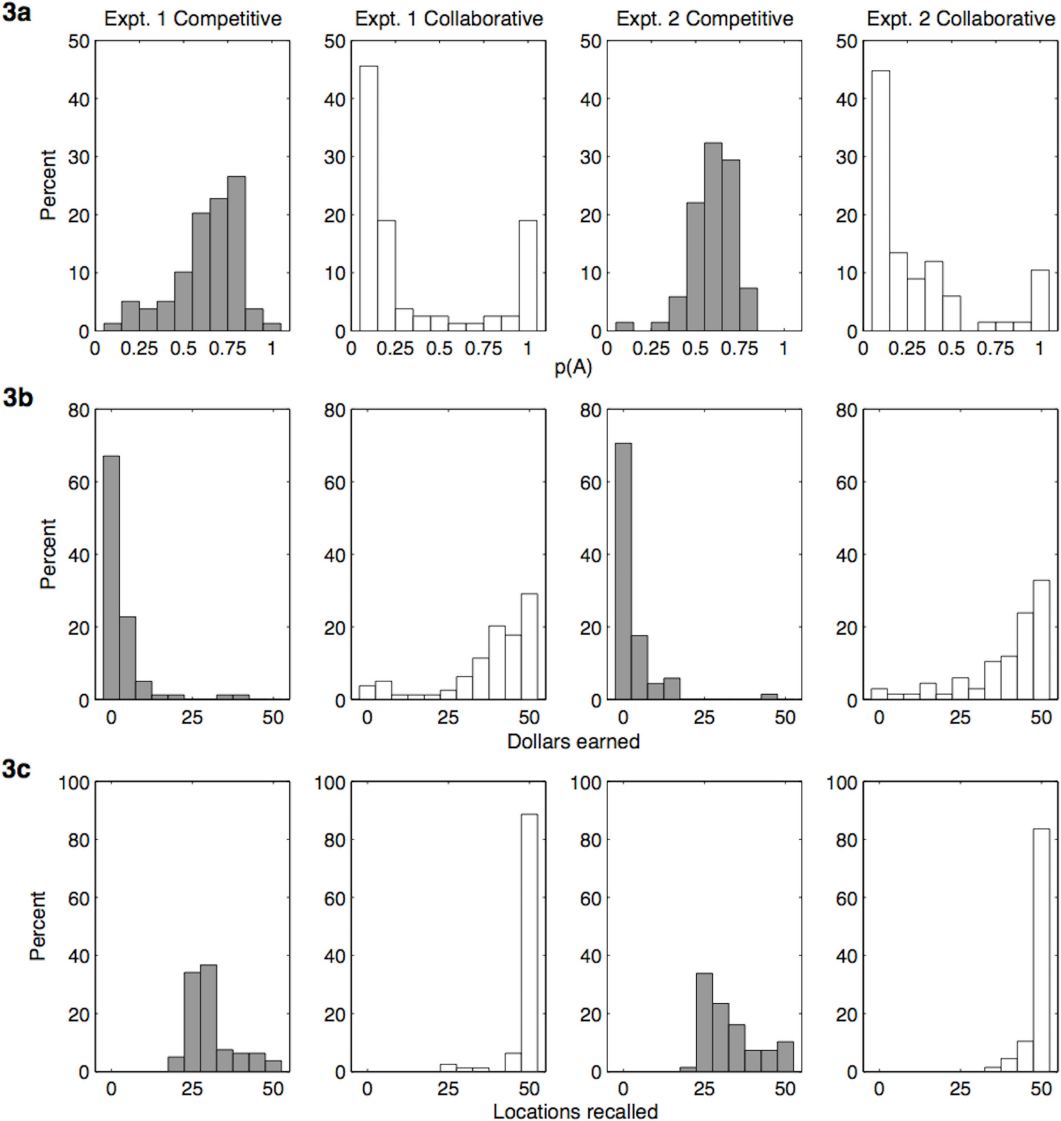
Hiding patterns, opponent search success, and hider recall search success by experiment and condition. Experiment 1: left-hand columns; Experiment 2: right-hand columns. Competitive condition: grey bars; collaborative condition: white bars. Distributions across participants of percent of (a) hiding patterns as measured by alternation rate *p*(*A*), (b) participants’ earnings in dollars for hiding, and (c) the number of locations participants could recall from their own earlier hiding patterns.

To test what type of resource distributions participants chose for hiding their dollar tokens from the other players in competitive and collaborative settings, we computed the overall alternation probability *p*(*A*) of each participant’s 2D grid pattern (by averaging the alternation rates computed row-wise and column-wise—see [25]). Fig 3A shows histograms of these alternation rates *p*(*A*) by experiment and collaborative versus competitive condition. Here we indicate the numeric results from Experiment 1 (which were qualitatively the same as those for Experiment 2 unless otherwise indicated). The differences in behavioral task variables were highly significant between conditions in both experiments (see Table 1 for details). We found no sex differences on the reported variables.

**Table 1:** Means and standard deviations of behavioral task variables, and comparison between competitive and collaborative conditions.

	Experiment 1 (*N* = 158)					
Task Variable	Competitive (*n* = 79)		Collaborative (*n* = 79)		*t*-test	
	*M*	*SD*	*M*	*SD*	*t*	*p*
*Hiding Task*						
Total completion time	182.27	110.27	102.36	74.72	5.33	< .001
Clicks to reach hiding pattern	67.24	30.09	64.48	40.95	0.48	.630
Probability of alternation *p*(*A*)	0.63	0.18	0.35	0.37	5.96	< .001
Configuration measure *C*	0.39	0.22	0.75	0.24	9.98	< .001
*Search Task*						
Total completion time	179.98	57.68	81.59	41.34	12.32	< .001
Clicks to find all dollars	96.62	6.81	62.08	13.77	19.99	< .001
Amount of dollars earned	3.38	6.81	37.92	13.77	-19.99	< .001
*Recall Task*						
Number of locations recalled	30.68	7.16	48.51	4.91	-18.24	< .001
	Experiment 2 (*N* = 135)					
Task Variable	Competitive (*n* = 68)		Collaborative (*n* = 67)		*t*-test	
	*M*	*SD*	*M*	*SD*	*t*	*p*
*Hiding Task*						
Total completion time	191.08	99.65	123.74	76.38	4.40	< .001
Clicks to reach hiding pattern	63.85	21.30	75.25	38.11	-2.15	.033
Probability of alternation *p*(*A*)	0.60	0.13	0.30	0.31	7.48	< .001
Configuration measure *C*	0.28	0.18	0.67	0.30	9.29	< .001
*Search Task*						
Total completion time	163.47	59.08	80.57	60.42	8.06	< .001
Clicks to find all dollars	96.79	6.85	60.85	13.00	20.14	< .001
Amount of dollars earned	3.21	6.85	39.15	13.00	20.14	< .001
*Recall Task*						
Number of locations recalled	32.76	8.24	48.88	2.66	-15.24	< .001

*Note*. Completion times are shown in seconds.

**Fig 4:**
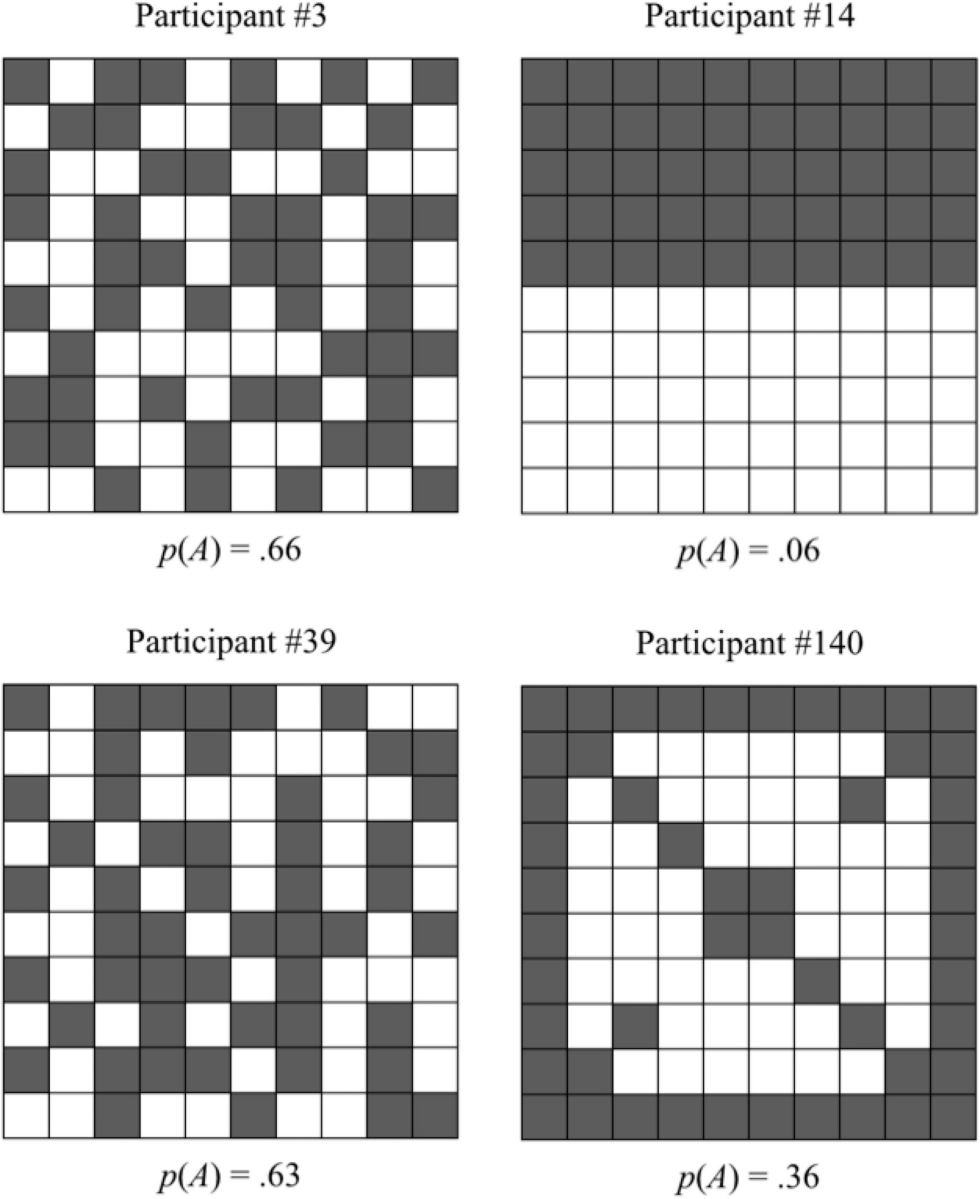
Sample resource grids created by participants in the hiding task. Plots show hiding patterns in Experiment 1 in the competitive condition (left side) and collaborative condition (right side).

As expected (Hypothesis 1), participants chose hyperdispersed resource distributions in the competitive setting [*p*(*A*) = .63]—see Fig 4 (left column) for examples. In the collaborative setting, participants mostly used clumpy hiding distributions [*p*(*A*) = .35], with many creating highly clustered patterns with very low alternation probabilities [for instance, 25 out of 79 participants in Experiment 1 created the single-clump pattern in Fig 4, top right, with *p*(*A*) = .06], supporting Hypothesis 2. However, a few collaborative participants (15 in Experiment 1 and 7 in Experiment 2) created perfectly alternating checkerboard patterns [i.e., *p*(*A*) = 1], presumably hoping that their opponents would look for resources that way, while others opted for resource displays showing aspects of symmetry [e.g., 11 in Experiment 1 used something close to Fig 4, bottom right, with *p*(*A*) = .36]. To capture the fact that grids with both very high and very low *p*(*A*) values are mostly highly patterned, we constructed a new configuration measure, *C* = |*p*(*A*) − .5|/.5, where values of *C* near 0 typically indicate a lack of pattern (randomness) and values near 1 indicate a strong pattern of blocks or checkerboard alternation. (Other rarer patterns are not characterized effectively by this measure, like that in the bottom right of Fig 4, but these are also more difficult for fellow searchers to figure out and exploit.) Using this statistic, we find that *C* = .39 on average in Experiment 1 in the competitive condition (.28 in Experiment 2), indicating an overall lack of pattern to make searching more difficult for the opponent, while *C* = .75 on average in Experiment 1 in the collaborative condition (.67 in Experiment 2), indicating greater overall use of block or alternation patterns to make searching easier for the opponent. The differences in relative absence or use of these patterns are statistically significant (see Table 1).

We can test how challenging it was for the hider to create the grid patterns they used by measuring the total number of locations they clicked on (either placing or deleting resources) and the total amount of time they took before moving on to the second stage of the experiment (where higher values on both measures indicate more effort). While participants in both the competitive and collaborative conditions used about the same number of clicks to create their final 50-token hiding pattern (67 vs. 64), participants in the competitive condition took significantly longer to decide how to hide their resources (182s vs. 102s). Across both conditions, higher alternation probabilities in participants’ hiding patterns (translating into more dispersed distributions) also correlated positively with the completion time in the hiding task (*r* = .25, *p* = .002), while higher patterning *C* made hiding quicker (*r* = -.32, *p* < .001). We replicated these results in Experiment 2.

When participants knew they would have to find the resources they hid in stage 1 at some later point (Experiment 2), they used slightly lower alternation rates [*p*(*A*) = .60 and .30 for competitive and collaborative conditions respectively, vs. .63 and .35 in Experiment 1], in keeping with Hypothesis 3. However, these differences between experiments were non-significant [competitive condition: *t*(145) = 0.99, *p* = .321; collaborative condition: *t*(144) = 0.96, *p* = .338]. These differences would probably be strengthened by increasing the importance of later recall, for instance by also paying participants for their performance on that part of the experiment (which we did not do in the present study).

### How successful were participants when searching?

As shown, participants searching for resources in the competitive condition where dollar tokens were mostly distributed in dispersed patterns had little success, earning only about $3 potential payoff on average (see Fig 3B). In contrast, participants searching in the collaborative condition had an easy time predicting the other player’s patterned resource distribution and received high potential earnings (about $38 on average). In Experiment 1, participants in the competitive condition needed more clicks (97 vs. 62 in the collaborative condition) and more time to reveal all 50 resources (180s vs. 82s). Across conditions, the alternation probability *p*(*A*) of their opponent’s hiding pattern correlated with their overall search time (*r* = .38, *p* < .001) and inversely with the payoff they ultimately earned (*r* = -.39, *p* < .001); the correlations with configuration measure *C* were even stronger [*r* = -.47, *p* < .001 and *r* = .70, *p* < .001, respectively; note that these correlations go in the opposite direction from those with *p*(*A*)]. Thus hiders overall were effective in creating patterns that were either easy or difficult for opponents to search through as the task required. The results in Experiment 2 were qualitatively the same (Table 1). No sex differences were found.

### Can participants recall the locations of resources they previously hid?

At the end of each experiment, participants were asked to recall the 50 locations where they hid their resources (by clicking on 50 out of the 100 possible spots). Participants just randomly guessing would locate about 25 dollar tokens by chance in this recall task. Participants did significantly better than chance in both conditions [Experiment 1, competitive condition: *M* = 31, *t*(78) = 7.06, *p* < .001; collaborative condition: *M* = 49, *t*(78) = 42.54, *p* < .001], but they had a much easier time overall in the collaborative condition, with most achieving perfect or near-perfect recall (see Fig 3C). Consistent with these results, the alternation probability *p*(*A*) of a hiding pattern strongly correlated with recall time (*r* = .43, *p* < .001) and number of resource locations that were recalled (*r* = -.45, *p* < .001), as did configuration measure *C* (*r* = -.48, *p* < .001 and *r* = .62, *p* < .001, respectively). Being told in Experiment 2 that they would have to recall their hiding locations later did not result in a significant improvement in participants’ recall [competitive condition: 33 locations vs. 31 locations in the surprise recall in Experiment 1, *t*(145) = 1.64, *p* = .104; collaborative condition: 49 locations each, *t*(144) = 0.56, *p* = .578]. (Such perfect or near-perfect recall involves more than just remembering the alternation probability of the stored location pattern, of course—for instance, it could involve remembering “one-cell checkerboard pattern, with top-left square empty”—but using extreme values such as *p*(*A*) = 1 lessens the memory load.) There were no differences in recall performance by sex.

### Do participants search differently in competitive and collaborative conditions?

**Fig 5:**
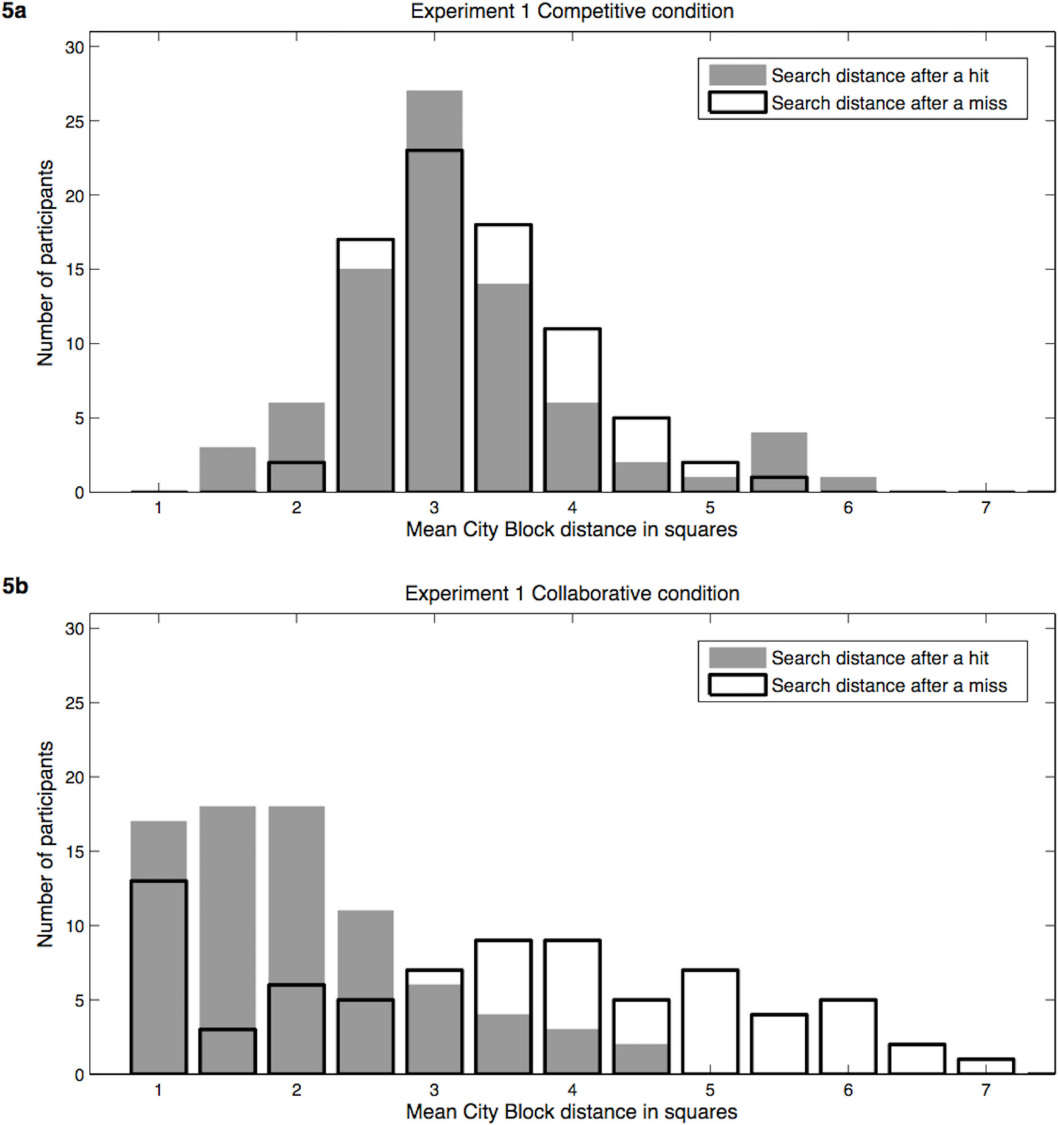
Distribution of each participant’s mean search distance after successfully finding a token (a hit) or clicking on an empty location (a miss). (a) Experiment 1 competitive condition, (b) Experiment 1 collaborative condition.

To test the degree to which participants in the two conditions searched for resources in a win-stay lose-shift manner appropriate for clumpy distributions, we analyzed how far they moved from the current search location to the next after finding versus not finding a resource at the current location. Participants searching for resources (hidden by others) during the second stage of each experiment received two kinds of feedback: success by finding a dollar token (i.e., a hit, or “win”) or failure by unveiling an empty location (i.e., a miss, or “lose”). A win-stay lose-shift strategy would result in a lower mean city-block distance (number of squares up/down and left/right) between the current and next clicked location after finding a resource (i.e., staying nearby after winning) than after finding an empty location (i.e., shifting further away after losing). In the competitive condition (Fig 5A) the distance moved was only slightly lower after a hit/win (3.15 squares) compared to a miss/loss (3.29 squares; *t*(78) = -1.45, *p* = .152), indicating that participants did not appear to use a win-stay lose-shift search strategy when they expected a challenging competitive situation (i.e., a random or hyperdispersed resource pattern, as indicated in the next section). In the collaborative condition (Fig 5B), however, participants tended to stay closer to the previous location after a hit (2.03 squares), helping them to continue to exploit the current patch, and significantly increased how far they moved after a miss (3.52 squares), helping them to raise their chance of hitting upon another resource patch elsewhere [*t*(78) = 66.36, *p* < .001]. These results support Hypothesis 4, showing sensitivity on the part of seekers to the environment structure they should expect in competitive versus collaborative settings. As before, Experiment 2 revealed qualitatively similar results, and there were no sex differences in either experiment.

### Do participants report using particular hiding and searching strategies?

We analyzed participants’ written responses about their strategy use during the hiding and searching parts of the experiment (see Table 2). Responses were coded based on whether they mentioned aggregation (e.g., “I tried to clump the bills together and leave large spaces with no bills”), randomness (e.g., “I hid them randomly and spread them out”), or dispersion (e.g., “every other square”). All responses that were left blank or were unclassifiable were coded as “other”. In the collaborative condition, the mode of the classifiable responses to all three questions in both experiments (ranging from 36–76%; see Table 2) was aggregation, highlighting participant’s preference for and expectation of patchy resource distributions in the absence of pilferers (cf. [2]). In the competitive condition, however, the categories with the highest percentages of classifiable coded responses shifted to randomness and dispersion (when asking what pattern participants created for their opponent). Interestingly, although the large majority of participants in the competitive condition hid their resources in hyperdispersed patterns [*p*(*A*) > .6 as reported above], an appreciable proportion of participants still reported thinking their opponents hid resources from them in a random distribution (22% in Experiment 1, 39% in Experiment 2) or even a patchy (aggregated) distribution (24%, 8%). This is consistent with previous findings that people tend to interpret moderately dispersed patterns as random, and to expect patchy patterns generally [2,3,25].

**Table 2:** Qualitative questionnaire data on hiding patterns used.

	Strategy Coding			
Strategy Question	Aggregation	Randomness	Dispersion	Other
Experiment 1				
*How did you decide to hide the tokens from the other player?*				
Competitive	16.5%	36.7%	21.5%	25.3%
Collaborative	50.6%	3.8%	2.5%	43.1%
*Did you try to create a particular pattern with the tokens*?				
Competitive	3.8%	16.5%	62.0%	17.7%
Collaborative	36.7%	0.0%	12.7%	50.6%
*Do you think your opponent used a particular strategy to hide the tokens from you*?				
Competitive	24.1%	22.8%	6.3%	46.8%
Collaborative	40.5%	2.5%	1.3%	55.7%
Experiment 2				
*How did you decide to hide the tokens from the other player*?				
Competitive	10.3%	32.4%	22.1%	35.3%
Collaborative	70.1%	1.5%	0.0%	28.4%
*Did you try to create a particular pattern with the tokens*?				
Competitive	8.8%	11.8%	11.8%	67.6%
Collaborative	76.1%	0.0%	0.0%	23.9%
*Do you think your opponent used a particular strategy to hide the tokens from you*?				
Competitive	8.8%	39.7%	11.8%	39.7%
Collaborative	71.6%	6.0%	1.5%	20.9%

*Note*. Percentages show the number of strategy question responses that were coded as mentioning aspects of aggregation, randomness, or dispersion; responses that were either blank or not classifiable are shown in the rightmost column (Other).

The results in the fourth stage of each experiment were also consistent with those previous findings. Here participants rated three sets of three 2D grids with alternation rates *p*(*A*) = .3, .5, or .7 in terms of which grid would be hardest to find resources in, which would be easiest, and which appears most random. As shown in Table 3, across experiments and conditions, participants thought of the hyperdispersed resource grid *p*(*A*) = .7 as both the hardest to search in and most random, while the aggregated (clumpy) pattern *p*(*A*) = .3 was seen as the easiest and least random. Interestingly, participants in the collaborative condition, most of whom made and searched through highly aggregated patterns, showed more extreme difficulty ratings for the hyperdispersed patterns and easy ratings for the aggregated patterns than participants in the competitive condition. There were no pattern rating differences by sex.

**Table 3:** Ratings of difficulty of 2D grid patterns.

	Chosen Grid Pattern		
Rated Dimension	Aggregated	Random	Dispersed
Experiment 1			
*Competitive*			
Hardest	26.6%	26.6%	46.8%
Easiest	57.0%	19.0%	24.0%
Most Random	11.4%	21.5%	67.1%
*Collaborative*			
Hardest	12.7%	17.7%	69.6%
Easiest	75.9%	6.3%	17.8%
Most Random	5.1%	21.5%	73.4%
Experiment 2			
*Competitive*			
Hardest	16.2%	27.9%	55.9%
Easiest	66.2%	13.2%	20.6%
Most Random	2.9%	33.8%	63.2%
*Collaborative*			
Hardest	11.9%	13.4%	74.6%
Easiest	80.6%	9.0%	10.4%
Most Random	4.5%	17.9%	77.6%

*Note*. Percentages show participants’ choices between an aggregated [*p*(*A*) = .3], random [*p*(*A*) = .5], or hyperdispersed [*p*(*A*) = .7] 2-dimensional grid pattern when asked to rate which appears hardest, easiest or most random to search in.

## Discussion

What spatial patterns do people expect when resources have been hidden so that they are easy or difficult to find? Here we addressed this question in terms of strategic foraging behavior via a sequential multi-person game in which participants hid resources for the next participant to try to find, either collaboratively or competitively. As predicted, we found that the type of interaction between players had a strong influence on hiding and searching behavior. When collaborating, resources were mostly hidden in clumpy distributions as are commonly seen in the natural world, but when competing, resources were hidden in more dispersed (seemingly random) patterns to increase the searching difficulty for the other player. More dispersed resource distributions came at the cost of higher overall hiding (as well as searching) times, decreased payoffs, and an increased difficulty when having to recall earlier hiding locations at the end of the experiment. Whether that later recall came as a surprise or was expected had no particular effect on the type of resource distributions participants chose. In line with research on area-restricted search mechanisms in animals, participants’ search strategies were affected by their underlying expectations for when resource distributions would be clumpy: When collaborating, participants appeared to use a win-stay lose-shift strategy appropriate to clumpy resources, but when competing, they moved similarly far after finding or not finding resources, as more appropriate for a random (non-clumped) resource pattern.

We found no significant sex differences in our data. Some previous studies have reported differences between the spatial search abilities of men and women, as predicted from an ancestral sexual division of labor, in object location tasks [56] and spatial navigation [57–59], while others have not (for visual search in [60]; object location memory in [59]). Whether the spatial search strategies and hiding-place recall abilities called upon in our study evoke the same foraging adaptations that others have investigated must be determined before our lack of sex differences can be compared to the results found by others.

We did not find much support for our third hypothesis that people would change their hiding strategy when they had to find the same resources again later. This could be because we only manipulated how much participants should pay attention to where they hid resources by either telling or not telling them that they would have to find the resources again later. A stronger manipulation for future work could include paying participants depending on how many of their own hidden resources they find again (a form of self-collaborative search), adding onto the payment for successfully hiding resources from others. Such studies could also investigate the relationship between recall time and choice of distribution for the hidden resources: Our preliminary data suggests that recall times in the retrieval stage of the study are longer for competitive condition patterns than collaborative ones, pointing to greater cognitive costs involved in competitive hiding and retrieving. Other experimental designs could allow participants to only search later for whatever resources they had hidden earlier that another searching participant did *not* find, which would be closer to foraging situations in the wild, at least for scatter-hoarding species; for humans who may not have faced such challenges, there may not be any specialized memory mechanisms for this kind of retrieval task, and instead they may rely on more consciously-applied general purpose memory strategies (e.g., making idiosyncratically memorable patterns).

## Conclusion

Human ancestors evolved in environments with resources spread out in various patterns, some made difficult to find, and others easier, depending on the adaptive goals of the agents involved in creating those resources (cf. [61,62]). Expectations of those resource patterns, whether clumpy in collaborative settings or dispersed in competitive ones, drive aspects of human searching behavior to this day—and can even come out in a simple game of hide and seek.

## Supporting Information

S1 Data Experimental data to experiment 1 and 2 (compressed zip archive). (ZIP)
